# Supplementary material for: Membrane insertion mechanism of the caveola coat protein Cavin1
Source: Proc Natl Acad Sci U S A. 2022 Jun 13;119(25):e2202295119. doi: 10.1073/pnas.2202295119 (PMC9231606; doi:10.1073/pnas.2202295119)
Supplement: Supplementary File [file pnas.2202295119.sapp.pdf]

Supplementary Material for

Membrane insertion mechanism of the caveola coat protein Cavin1

Kang-Cheng Liu[a], Hudson Pace[a,b,c], Elin Larsson[a], Shakhawath Hossain[d], Aleksei Kabedev[d], Ankita Shukla[a], Vanessa Jerschabek[e], Jagan Mohan[a,1], Christel A. S. Bergström[d], Marta Bally[b,c], Christian Schwieger[e], Madlen Hubert[a,d,2] and Richard Lundmark[a,2]

Affiliations

a. Integrative Medical Biology, Umeå University, 901 87, Umeå, Sweden.

b. Department of Clinical Microbiology, Umeå University, 901 85, Umeå, Sweden.

c. Wallenberg Centre for Molecular Medicine, Umeå University, 901 85, Umeå, Sweden.

d. Department of Pharmacy, Uppsala University, Uppsala Biomedical Center, 751 23 Uppsala, Sweden.

e. Institute of Physical chemistry, Martin Luther University Halle-Wittenberg, 06120 Halle (Saale), Germany.

1. Current address: Membrane Biochemistry and Transport, Institute Pasteur, 28 rue du Dr Roux, 75015 Paris, France.

2. Corresponding authors: Richard Lundmark, Department of Integrative Medical Biology, Umeå University, 901 87 Umeå, Sweden, email: richard.lundmark@umu.se.
Madlen Hubert, Department of Pharmacy, Uppsala University, Uppsala Biomedical Center, 751 23 Uppsala, Sweden, email: madlen.hubert@farmaci.uu.se.

This PDF file includes:

Supplementary Materials and Methods including computational simulation programs, IRRAS analysis, scripts and related references.

Figs. S1 to S7.

Supplementary Materials and Methods

IRRA spectra simulation, band fitting parameters and principal component analysis.

IRRA spectra of the protein containing monolayer recorded on a D_2O subphase (Sigma) were simulated in the range of the amid I' vibration according to a three-layer model reported by Kuzmin et al. (1). Simulation and fitting of the multicomponent IRRA bands were performed as described in Schwieger et al. (2). The optical constant of the subphase D_2O were taken from Berti et al. (3). The refractive index of the lipid/polymer film was set to 1.41 and its layer thickness to 2 nm. The band positions of the subcomponents were derived from second derivative spectra and revealed the contribution of three amide I' band components centered at 1662, 1642, and 1626 cm^{-1}. These components were assigned to one random and two α-helical secondary structure elements, respectively (4). According to literature, the lowest component could also be related to β-sheet amid I' vibrations (5). However, neither the X-ray structure nor CD spectra gave any indication for the presence of β-sheet components in the structure of Cavin1. Rather, we assigned the two lower spectral components to water exposed and water shielded faces of the helices, respectively, as reported for other coiled-coil helical arrangements (6). In analogy, we assumed that the amide bonds in helical structures facing the aqueous subphase are more water accessible and absorb at lower wavenumbers (1626 cm^{-1}), whereas the interaction with lipid monolayer reduces hydrogen bonds with hydration water leading to a shift to higher wavenumbers (1642 cm^{-1}). The band component assigned to amide I' vibrations of unordered structures (1662 cm^{-1}) was assumed to originate from isotropically distributed amid bonds and, therefore, simulated with an order parameter of $S = 0$ (corresponding to a tilt angle θ of 54° with respect to the interface normal). The polar angle between the helix main axis and the transition dipole moments of the helix amid I' vibrations was set to $\alpha = 38°$ (5). The main axis tilt angle of the two lower band components were set to be identical and varied in a least square Levenberg–Marquardt fit to determine the most probable helix orientation. Further fitting parameters were the full-widths at half-height (*fwhh*) of the three band components as well as the respective absorption coefficients k_{max}. The spectra were fitted in the range of 1610–1670 cm^{-1} and at angles of incidence, $\varphi = 30–70°$ in increments of 4°, where $\varphi = 49, 52, 55$ and 58° were omitted from the fit, because of low reflectivity in the range of the Brewster angle. For better understanding and coherence with the MD simulation results, the determined tilt angles θ, which are defined with respect to the interface normal, are translated into

inclination angles γ, which are defined in relation to the plane of the interface (γ = 90°-θ). The confidence interval of the fit minimum was calculated as follows: the goodness of the fit was assessed as the sum of square deviations at each inclination angle γ (SSD). These values were weighted by their minimum with (N-p), were N = 360 is the number of fitted data points and p = 6 is the number of fitted parameters:

$$X = \frac{SSD - SSD_{min}}{SSD_{min}} - 1 \times (N - p)$$

The X values are F-distributed with 1 degree of freedom. In the given conditions all $X \leq 3.87$ ($SSD \leq 1.26 \cdot 10^{-5}$) are within the 95% confidence interval.

A set of IRRA spectra was subjected to principal component analysis (PCA) in order to identify subtle changes in the band shape correlated to hydration differences. The PCA was performed on vector-normalized spectra (to exclude contributions from intensity variations to the principal components) in the range of 1693–1770 cm^{-1}. All spectra were pretreated as follows: i) a reference spectrum of a bare D_2O subphase was subtracted from all spectra, ii) a D_2O vapor spectrum was subtracted in a way to best reduce the contribution of vibrational-rotational bands in the spectral region of interest, iii) a polynomial rubber band baseline was subtracted from the spectra (Software OPUS, Bruker, Germany). All analyzed spectra were recorded in s-polarization, at various angles of incidence, φ. Four sets of spectra were analyzed in a common PCA: i) and ii) spectra of a DOPC:DOPE and of a DOPC:DOPE:PI(4,5)P_2 monolayer before injection of protein (20 mN m^{-1}, s-polarization, φ = 40°) and ii) and iv) spectra of the respective monolayers after injection of Cavin1 (1-190) (*ca.* 36 mN m^{-1}, s-polarization, φ = 25–70°). The PCA was performed using the *princomp* function of MATLAB (Math-Works Inc., Natick, MA, USA).

Computational simulations programs and scripts

Coarse-grained molecular dynamics simulations of unrestrained protein. For the coarse-grained (CG) simulations, Martini 2.2 force field (7, 8) was used and the initial PDB structure ID

4QKV (9) was coarse-grained using the Charmm-GUI platform (10, 11). No restraint was added to the Martini coarse-grained MD model of the HR1 coiled coil. A similar Martini HR1 domain was utilized in an earlier study (12) where the 4QKV atomistic structure was coarse-grained by using the Martinize.py script (13). The CG lipid membrane was generated using the insane.py script (11). Each simulation consisted of 674 lipid molecules per leaflet. Two different membrane compositions with molar ratio of DOPC:DOPE:PI(4,5)P_2 (55:40:5) and DOPC:DOPE (60:40) was used in different simulations. The topology of different lipid molecules was obtained from the martini website (http://cgmartini.nl/). The initial simulation box dimensions were 21, 21, and 27 nm in the x, y, and z directions, respectively. Each simulated system was first energy minimized using the steepest descent algorithm. This was followed by four short equilibration runs (100,000 steps) with time steps of 1, 2, 5, and 10 fs, before the final production run. The Verlet cutoff scheme was used for both the Coulombic (reaction-field) and van der Waals interactions with a 1.1 cutoff value. The v-rescale scheme (tau-t = 1 ps) was used for the thermostat. The final production run was performed using 15 fs time steps and the temperature was set at 37°C. Pressure coupling during equilibration was performed using the Berendsen barostat with tau-p = 5 ps, compressibility = $3e^{-4}$ bar^{-1}, ref-p = 1.0 bar for semi-isotropic systems. For the final production run, the pressure coupling was switched to Parrinello−Rahman with tau-p = 12 ps, compressibility = $3e^{-4}$ bar^{-1}, ref-p = 1.0 bar for semi-isotropic systems. The final production run was performed using 15 fs time steps and the temperature was set at 37°C. A periodic boundary condition was applied for all simulations with semi-isotropic pressure coupling due to presence of lipid membrane in the system. An in-house python code was used to calculate the distance between membrane center and the nearest residue from the membrane center. The number of contacts between the membrane lipid molecules and protein helices was obtained using the 'gmx mindist' tool in Gromacs with a cutoff distance of 0.6 nm for each component. All MD simulation snapshot images were generated using the molecular visualization program VMD (14). Images representing the electrostatic surface potential of the HR1was generated using PyMOL.

To calculate the membrane thickness, we have utilized the approach used by Buchout et al. (15), Briefly in this approach, a local normal vector is calculated from the lipid headgroup bead to the centroid of the lipid for each lipid molecule. Then to calculate the thickness, for each lipid headgroup bead the surrounding lipid headgroup beads were first determined (Fig. S7C). Then

from the average position of the selected neighbors, a second neighbor search is performed to identify the lipids which are in the other leaflet. The average position is calculated from the selected neighbors in the other leaflet as well. Based on the two average positions at the upper and lower leaflets and the local normal of the average position of the upper leaflet, the inter leaflet distance is estimated (Fig. S7C). This method is implemented in the opensource code FATSLiM (https://pythonhosted.org/fatslim/index.html). For each of the lipids, the position and thickness or the inter leaflet distance can be obtained using the code FATSLiM.

To calculate the distance between the membrane center and amino acids, we first select the amino acid beads of the protein for which we aim to calculate the beads distance. Then we determine the surrounding lipids molecules of that amino acid bead. The average positions of those surrounding lipid molecules and their thickness or inter leaflet distance was then obtain using the code FATSLiM. These values were then utilized to identify the membrane center position. The distance between the membrane center and amino acid beads were then calculated. At a specific simulation time, the minimum distance, d_{min}, was calculated as the distance between the membrane center and the center of the nearest bead of any protein residues. This d_{min} was calculated every 10 ns and the average minimum distance, $\langle d_{min} \rangle$, represents the average of all the d_{min} values.

Hydrogen bonds and protein-membrane interactions in all-atom molecular dynamics simulations. All-atom simulations were performed in order to investigate the formation of the hydrogen bonds between the Cavin1 HR1 (unrestrained) and the membrane. The simulations were performed using CHARMM36m force field (16) in triplicate. The position of the protein, as well as the number of hydrogen bonds formed between the protein and the membrane (including more specific helices-membrane and lipids-protein bonds) were evaluated. The “gmx hbond” module was used to evaluate the number of hydrogen bonds formed between different groups of molecules. The all-atom simulations were equilibrated and followed by a production run lasting 300 ns. The latter was performed using 2 fs time step. Verlet cutoff scheme was used with the radii of 1.2 nm for both coulomb and van der Waals interactions. PME type of electrostatic interactions representation was applied. Force-switch modifier was also applied to the van der Waals interactions, with a radius of 1.0 nm. Nose-Hoover thermostat was used with a τ_t=1.0. Parrinello-Rahman barostat with semi-isotropic coupling was used with the τ_p=5.0, compressibility of 4.5×10^{-5} and reference pressure of 1 atmosphere. LINCS algorithm was applied to hydrogen bonds.

Table 1. List of all performed MD-simulations.

**Simulation description**	**Components with number of molecules**	**Simulation time**	**# of simulations**
HR1 Trimer with membrane	DOPC – 742, DOPE – 540, PI(4,5)P_2 – 66, water beads – 84000, HR1 trimer – 1, Sodium ion – 330, Chloride ion – 6	2 μs	6
		36 μs	3
HR1 Trimer with membrane without PI(4,5)P_2	DOPC – 810, DOPE – 540, water beads – 84000, HR1 trimer – 1, Chloride ion – 6	2 μs	3
Helix A of the HR1 Trimer with membrane	DOPC – 742, DOPE – 540, PI(4,5)P_2 – 66, water beads – 84000, Helix A of the HR1 trimer – 1, Sodium ion – 330, Chloride ion – 3	2 μs	3
HR1 Trimer in water without membrane	Water beads – 84000, HR1 trimer – 1, Chloride ion – 6	36 μs	2
AA simulations of the HR1 on the top of the membrane	DOPC – 484, DOPE – 352, PI(4,5)P_2 – 44, water molecules – 120609, HR1 trimer – 1, Sodium ion – 492, Chloride ion – 331 (number of water molecules differed between the simulations)	0.3 μs	3

Supplementary figures

Figure S1

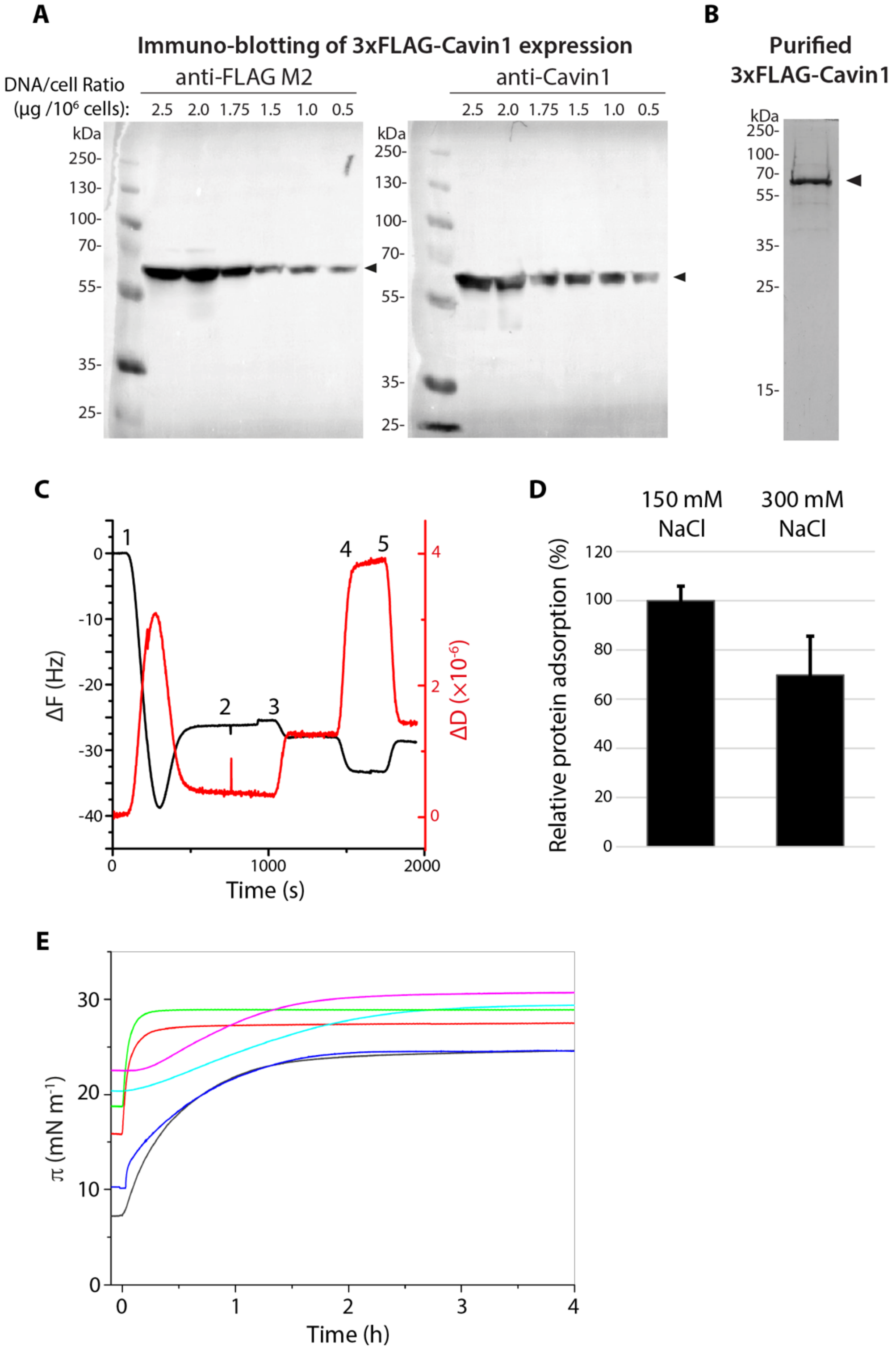

Fig. S1. Purification of full-length Cavin1 expressed in HEK 293-F cells and monolayer adsorption experiments. (A) Immunoblot of lysates from HEK 293-F cells transfected with different amount of 3×FLAG-Cavin1 expression plasmid as indicated. **(B)** Coomassie stained SDS-PAGE of purified 3×FLAG-Cavin1 protein. **(C)** QCM-D sensogram showing the effect of buffer exchanges without protein. 1) the adsorption of POPC:PI(4,5)P_2 vesicles in 20 mM citrate, 50 mM KCl, 0.1 mM EDTA (pH 4.5), 2) the completed POPC:PI(4,5)P_2 SLB at equilibrium still in 20 mM citrate, 50 mM KCl, 0.1 mM EDTA (pH 4.5), 3) the change to 20 mM HEPES, 150 mM NaCl (pH 7.4), 4) the change to 20 mM HEPES, 300 mM NaCl (pH 7.4), 5) the change back to 20 mM HEPES, 150 mM NaCl (pH 7.4). Note the shifts in frequency that occur with each buffer change, as though apparent mass is gained when changing to a buffer with higher salt concentration. Also note that this phenomenon is reversible. **(D)** Quantification of Cavin1 adsorption to SLBs as measured by QCM-D in buffer supplemented with 150 mM or 300mM NaCl. **(E)** Adsorption of Cavin1 to lipid monolayers. Lipid monolayers consisting of DOPC:DOPE:PI(4,5)P_2 (55:40:5 mol%) were prepared on a buffer subphase and Cavin1 was injected underneath the film at different initial surface pressure (π_0). The surface pressure (π) was recorded as a function of time. This data was used to determine the maximum insertion pressure (MIP) of Cavin1 shown in Fig. 1F.

Figure S2

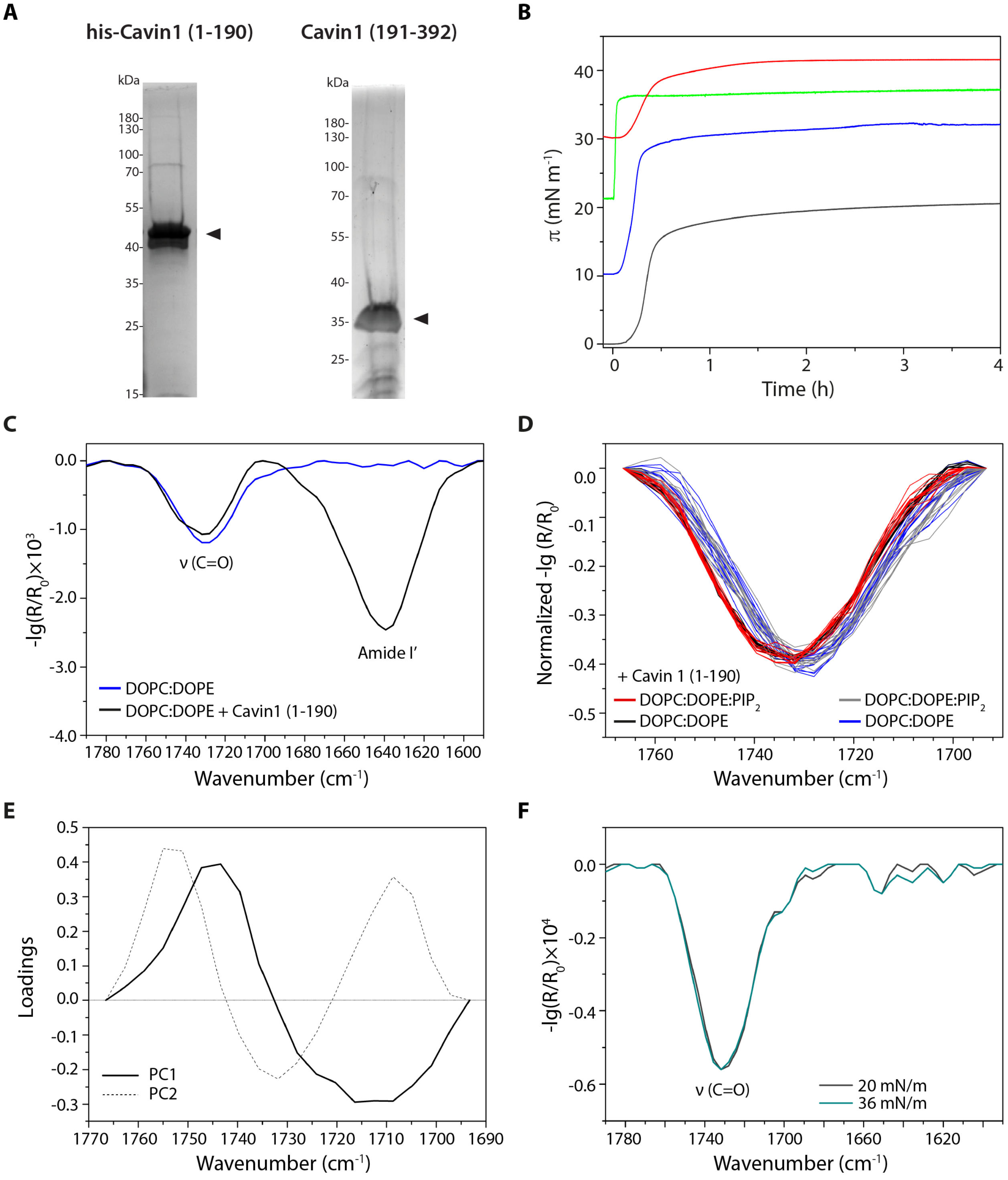
A
his-Cavin1 (1-190)
Cavin1 (191-392)
kDa
180-
130-
100-
70-
55-
40-
35-
25-
15-
kDa
180-
130-
100-
70-
55-
40-
35-
25-
B
π (mN m-1)
Time (h)
C
-lg(R/R0)×10^3
ν (C=O)
Amide I'
DOPC:DOPE
DOPC:DOPE + Cavin1 (1-190)
Wavenumber (cm-1)
D
Normalized -lg (R/R0)
+ Cavin 1 (1-190)
DOPC:DOPE:PIP2
DOPC:DOPE
DOPC:DOPE:PIP2
DOPC:DOPE
Wavenumber (cm-1)
E
Loadings
PC1
PC2
Wavenumber (cm-1)
F
-lg(R/R0)×10^4
ν (C=O)
20 mN/m
36 mN/m
Wavenumber (cm-1)

Fig. S2. Purification, monolayer adsorption and IRRAS analysis of Cavin1 (1-190). (A) Coomassie stained SDS-PAGE of purified Cavin1 (1-190) and (191-392) proteins from bacterial expression. (**B)** Adsorption of Cavin1 (1-190) to lipid monolayers. Lipid monolayers consisting of DOPC:DOPE:PI(4,5)P_2 (55:40:5 mol%) were prepared on a buffer subphase and Cavin1 (1-190) was injected underneath the film at different initial surface pressure (π_0). The surface pressure (π) was recorded as a function of time. This data was used to determine the maximum insertion pressure (MIP) of Cavin1 (1-190) shown in Fig. 2C. **(C)** IRRA spectra (1790–1590 cm^{-1}) of DOPC:DOPE (60:40 mol%) at an initial surface pressure of 20 mN m^{-1}. The characteristic C=O vibrational band at ~1730 cm^{-1} originates from lipid ester groups. The amide I' band with a maximum at ~1640 cm^{-1} indicates presence of Cavin1 (1-190) after its injection into the subphase. Spectra were acquired with p-polarized light at an angle of incidence of 40°. **(D)** Set of spectra used in the principal components analysis (PCA): vector normalized IRRA spectra in the C=O stretching vibrational region measured in s-polarization and at various angles of incidence of lipid monolayers prepared with and without PI(4,5)P_2 and in presence and absence of Cavin1 (1-190), respectively, as indicated. **(E)** Results of PCA of the spectra shown in (D): loadings (coefficients) of the first two principal components (PC1, solid line and PC2, dotted line), which represent 76% and 10% of the total variance of the data, respectively. PC1 is indicative for a shift of the band to lower wavenumbers and can be interpreted as an increase in hydration of the carbonyl groups. The scores of the individual spectra on PC1 and PC2 are shown in Fig. 2F. **(F)** IRRA spectra of pure lipid films of DOPC/DOPE/PI(4,5)P_2, without protein, at the surface pressure of 22 and 36 mN m^1. Spectra were acquired with p-polarized light at an angle of incidence of 40°.

Figure S3

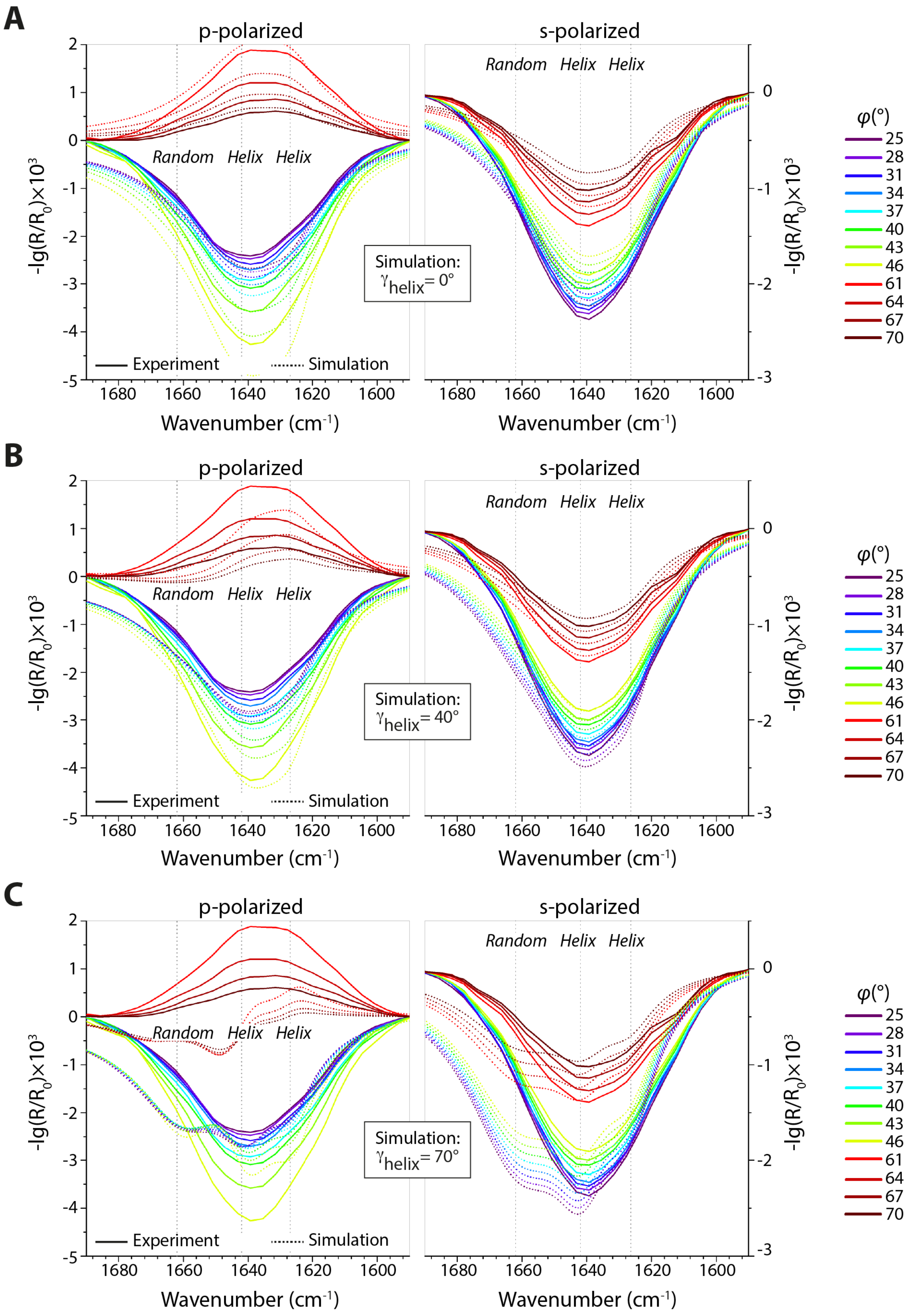
A
B
C
p-polarized
s-polarized
Random Helix Helix
Simulation: γ_{helix}= 0°
Simulation: γ_{helix}= 40°
Simulation: γ_{helix}= 70°
Experiment
Simulation
-lg(R/R$_0$)×10^3
Wavenumber (cm^{-1})
φ(°)
25
28
31
34
37
40
43
46
61
64
67
70

Figure S3

D

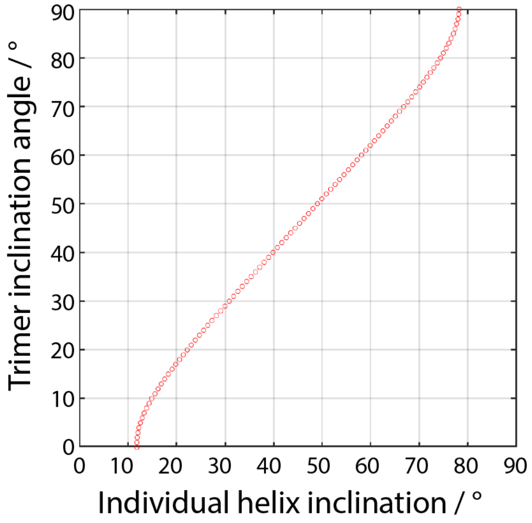

Fig. S3. Evaluation of spectral fits of IRRA amid I' bands outside the 95% confidence interval of the minimum. Experimental (solid) and best fitting simulated (dotted) IRRA spectra of Cavin1 (1-190) adsorbed to a DOPC/DOPE/PI(4,5)P_2 monolayer, recorded at various angles of incidence φ (see legend) and polarizations. The three band components used for simulation are indicated in the figure (vertical dotted lines). For SSD values of the shown fits see Fig. 3D. **(A)** The helix inclination angle was set to $\gamma = 0°$ which is identical to a completely parallel orientation of all helices to the lipid layer, which is only possible after HR1 uncoiling. Note that the shapes of the experimental IRRA amid I' bands could be reproduced by the fit but the intensities are not in perfect accordance. **(B)** The helix inclination angle was set to $\gamma = 40°$ (HR1 inclination: 40°). Note that the shape of the positive amid I' bands cannot be reproduced by the fit. **(C)** The HR1 inclination angle was set to $\gamma = 70°$, which is nearly upright (HR1 inclination: 74°). Note that that neither shape nor sign of the amid I' bands can be reproduced by the fit. **(D)** Relation between inclination angles of individuals helices and the trimeric helix bundle with respect to the interface. Note that in a flatly adsorbed helix bundle (trimer inclination angle = 0°) the individual helices still have a residual average inclination (12°), as they are wrapped around each other and cannot be completely stretched.

Figure S4

A

B

C

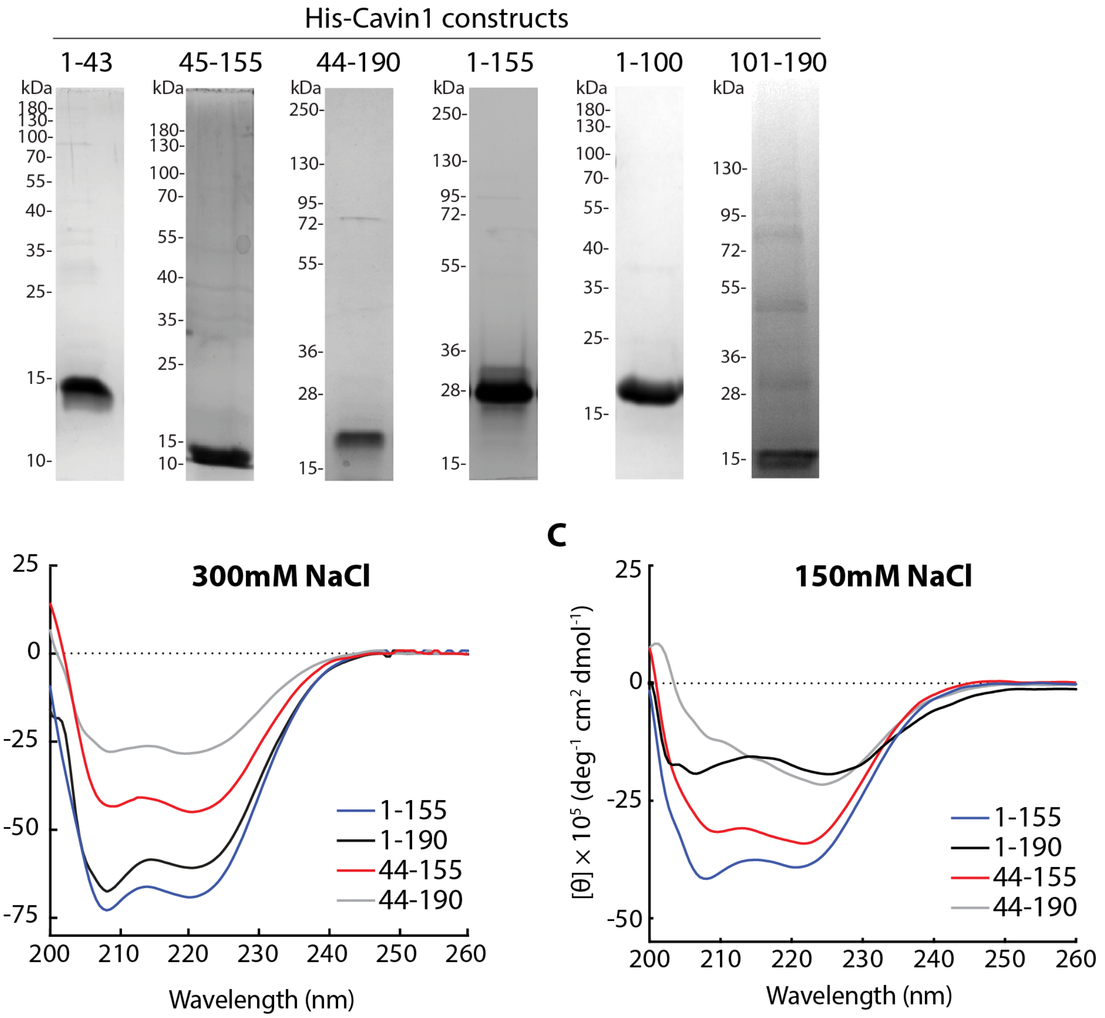

D

Protein	Mass (kDa)	σ	Molecule count (%)	Oligomerization state
**1-155**	55	18.3	3306 (109%)	trimer
**1-190**	32	8.6	481 (24%)	monomer
	100	13.5	1268 (63%)	trimer
**45-155**	48	13.2	1761 (96%)	trimer
**44-190**	55	12.0	1692 (75%)	trimer
	157	10.9	129 (6%)	nonamer
**1-100**	60	12.7	2469 (98%)	trimer
**101-190**	42	13.9	676 (55%)	trimer

Fig. S4. Purification, CD analysis and mass photometry analysis of different truncated Cavin1 proteins. (A) Coomassie stained SDS-PAGE gels of truncated Cavin1 proteins as indicated purified from bacteria: DR1, his-Cavin1 (1-43); HR1, his-Cavin1 (44-155); HR1+DR2, his-Cavin1 (44-190); DR1+HR1, his-Cavin1 (1-155), Cavin1 (1-100) and his-Cavin1 (101-190). **(B-C)** Far-UV CD spectra (195–255 nm) of Cavin1 constructs as indicated in 300 mM NaCl buffer **(B)** or 150 mM NaCl buffer **(C)**. **(D)** Table showing mass photometry characterization of different Cavin1 truncated mutants. The molecular mass was obtained by comparison to a BSA standard. The histograms of mass distribution were processed by Gaussian fitting, where σ indicates the standard deviation. Molecule counts show the number of fitted particles, and the percentage (%) in relation to all the detected particles in the experiments. The oligomerization state was calculated by dividing the measured particle mass by the predicted molecular mass of monomer based on amino acids sequences.

Figure S5

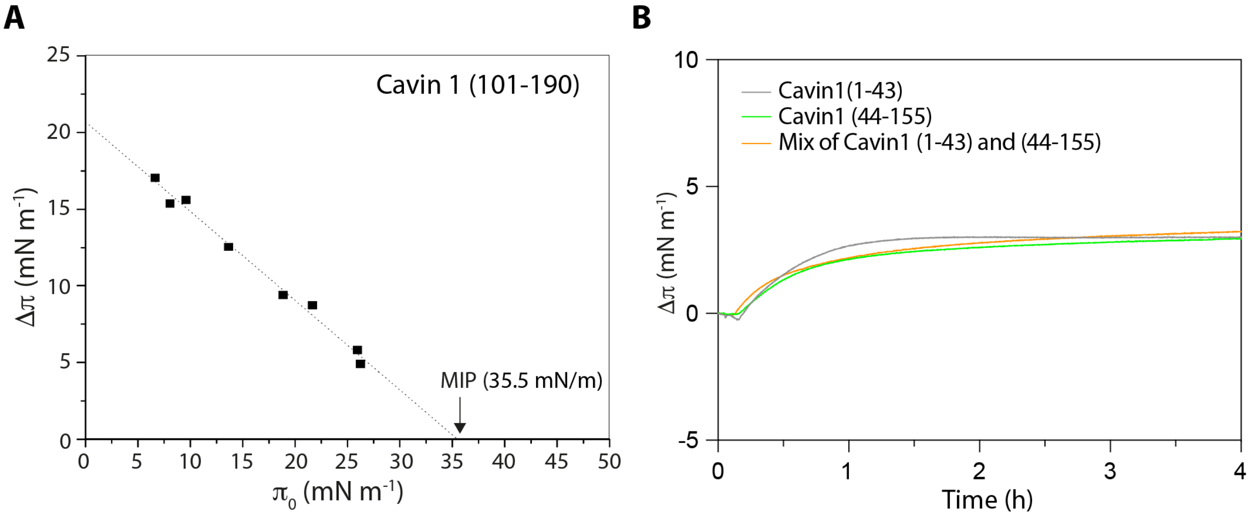

Fig. S5. Monolayer adsorption of different truncated Cavin1 proteins. (A) Cavin1 (101-190) adsorption to lipid monolayers was measured at different π_0. The MIP value was determined by extrapolation of the $\Delta\pi/\pi_0$ plot to the x-axis. **(B)** Adsorption of Cavin1 constructs to monolayer composed of DOPC:DOPE:PI(4,5)P_2 (55:40:5 mol%). Monolayers were prepared at π_0 = 20 mN m^{-1}. Following injection of the various truncates underneath the film, the surface pressure change ($\Delta\pi$) was recorded over time.

Figure S6

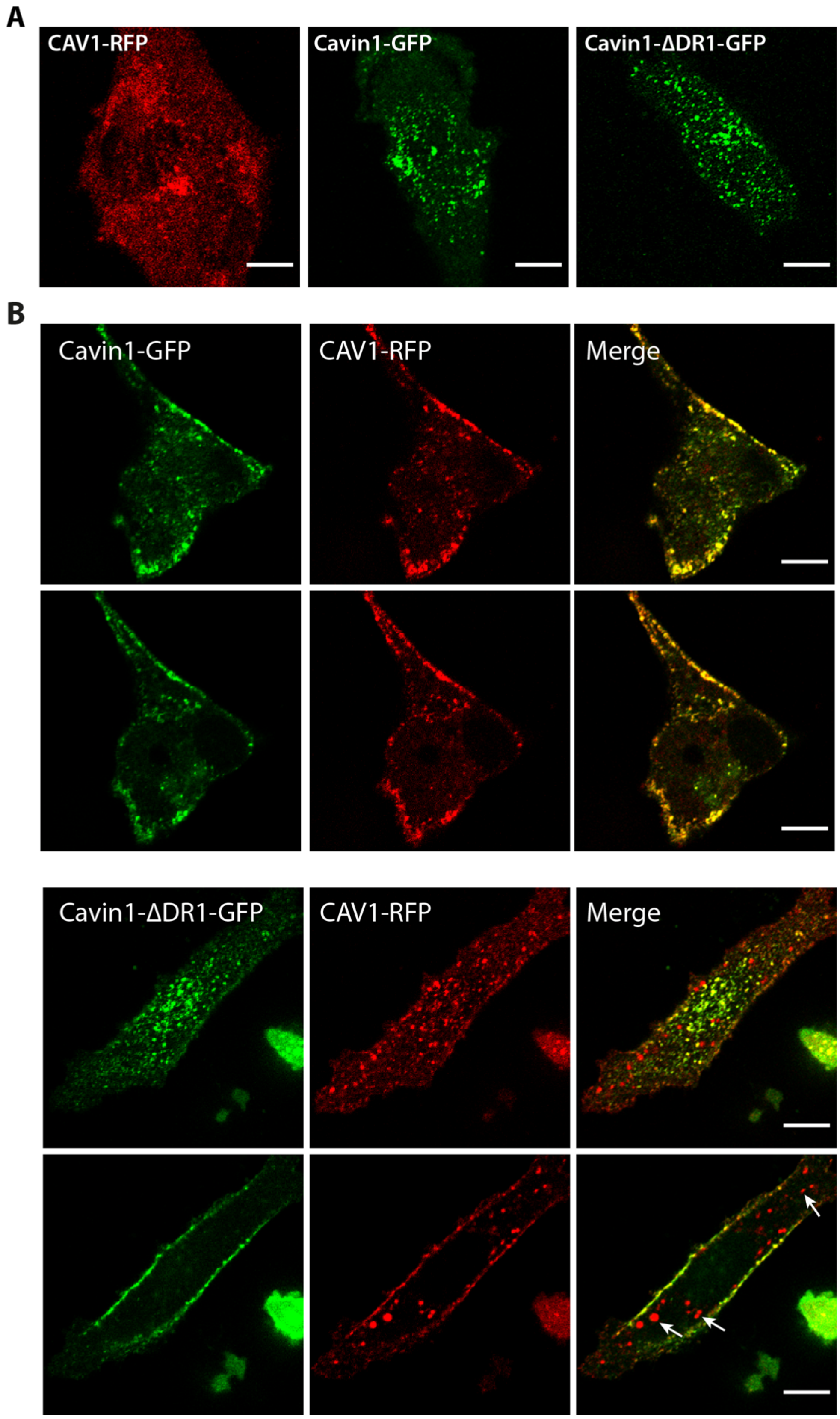
A
CAV1-RFP
Cavin1-GFP
Cavin1-ΔDR1-GFP
B
Cavin1-GFP
CAV1-RFP
Merge
Cavin1-ΔDR1-GFP
CAV1-RFP
Merge

Figure S6

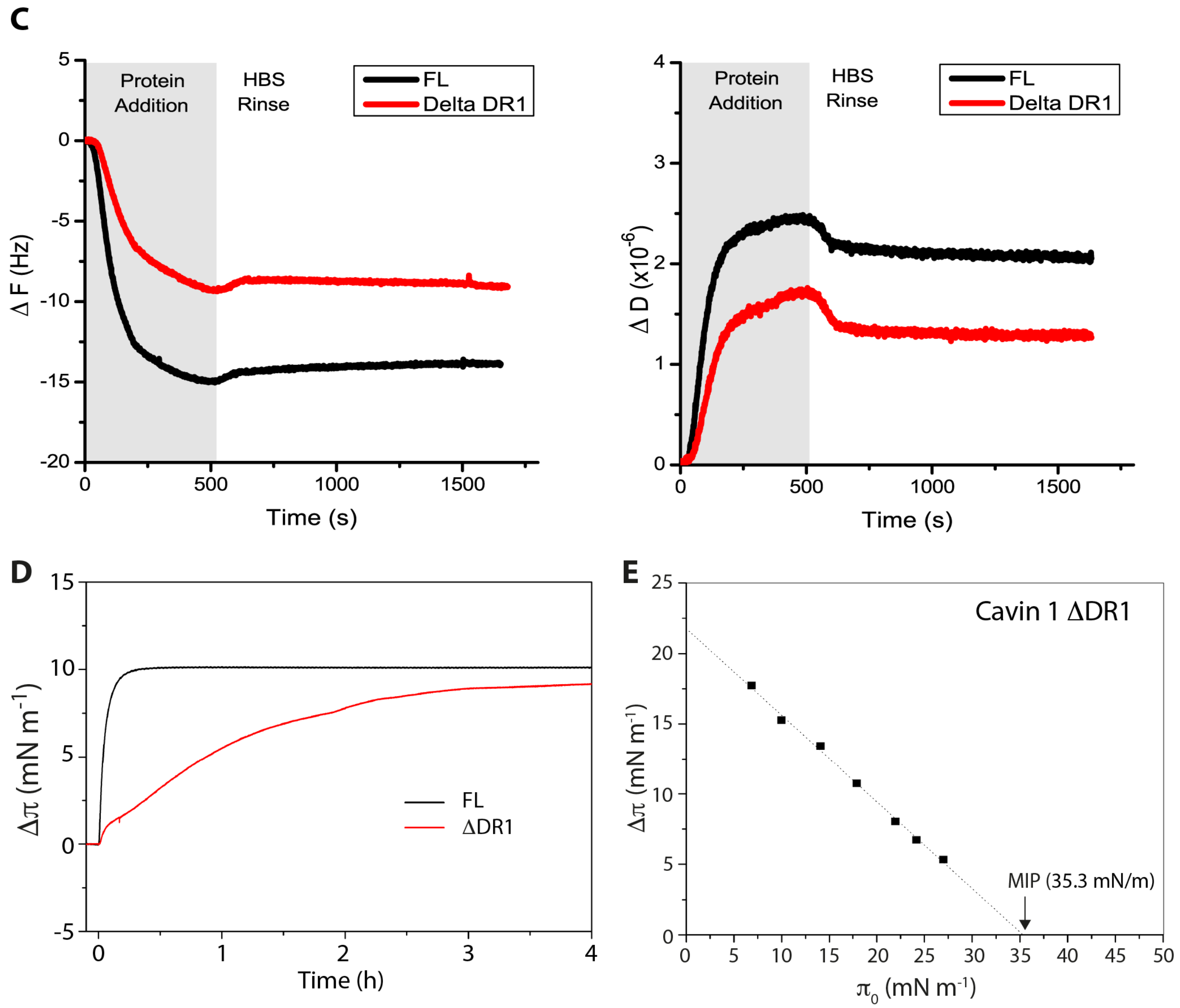

Fig. S6. Deletion of the DR1 hampers membrane binding and caveolae assembly in cells, but not membrane insertion. (A) Representative confocal microscopy images of PC-3 cells expressing CAV1-RFP, Cavin1-GFP and Cavin1-ΔDR1-GFP as indicated. **(B)** Representative confocal microscopy images of PC-3 cells transfected with fluorescently tagged CAV1 (CAV1-RFP, red), full-length Cavin1 (Cavin1-GFP, green) or Cavin1-ΔDR1 (Cavin1-ΔDR1-GFP, green). Focal plane in the top panel shows protein localization at the basal membrane and in the bottom panel protein localization in the mid-section of a cell. White arrows highlight intracellular CAV1 structures devoid of Cavin1-ΔDR1-GFP. Scale bar, 10 μm. **(C)** Frequency shift (ΔF) and Dissipation shift (ΔD) as a result of adsorption of ΔDR1 Cavin1 and full length Cavin1 onto SLBs measured by QCM-D. **(D)** Adsorption of ΔDR1 and full length Cavin1 to monolayer composed of

DOPC:DOPE:PI(4,5)P_2 (55:40:5 mol%). Monolayers were prepared at π_0 = 20 mN m^{-1}. Following injection of the various truncates underneath the film, the surface pressure change ($\Delta\pi$) was recorded over time. **(E)** Adsorption of ΔDR1 to lipid monolayers was measured at different π_0. The MIP value was determined by extrapolation of the $\Delta\pi/\pi_0$ plot to the x-axis.

Figure S7

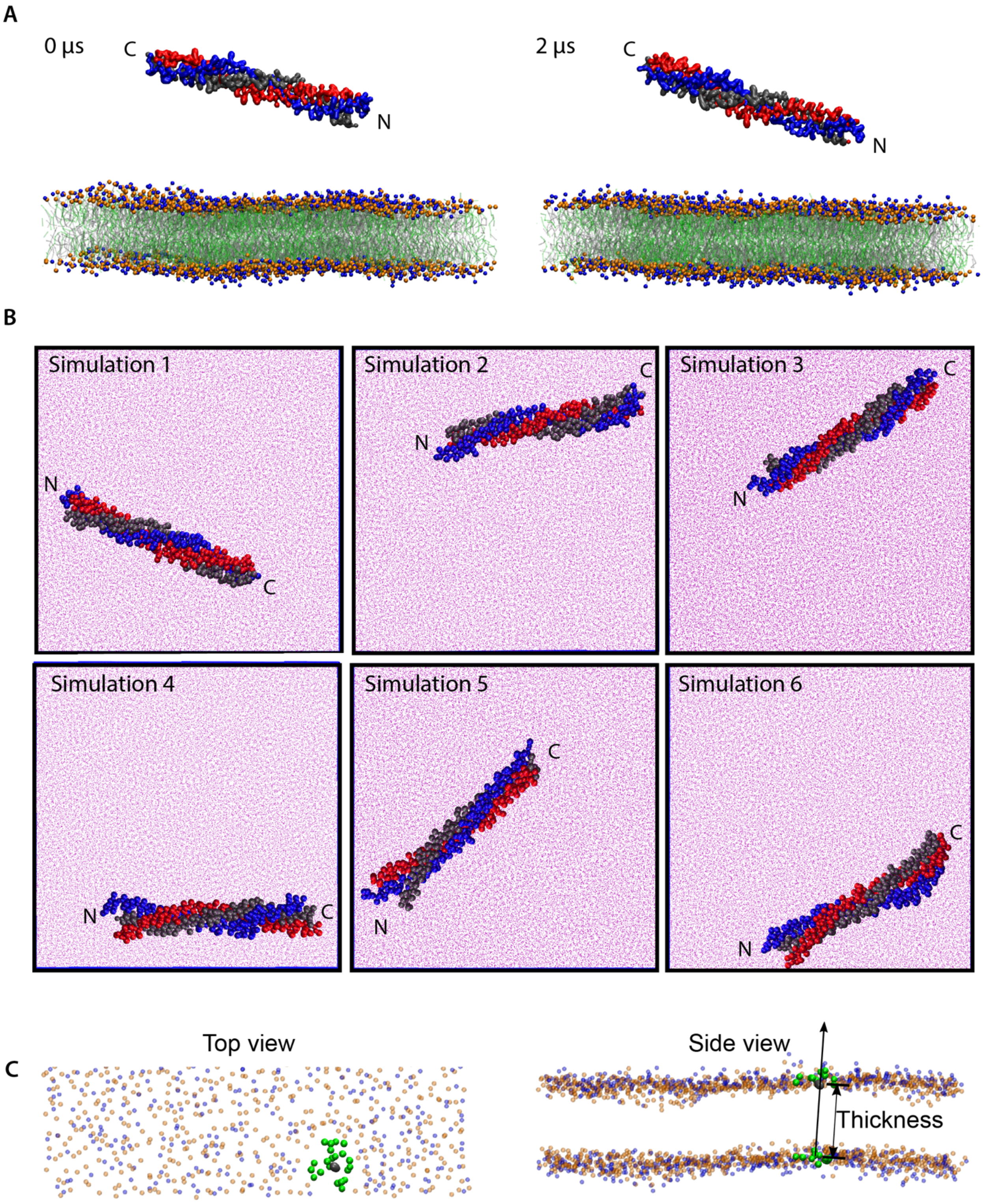
A
0 µs
C
N
2 µs
C
N
B
Simulation 1
N
C
Simulation 2
C
N
Simulation 3
C
N
Simulation 4
N
C
Simulation 5
C
N
Simulation 6
C
N
C
Top view
Side view
Thickness

Figure S7

D

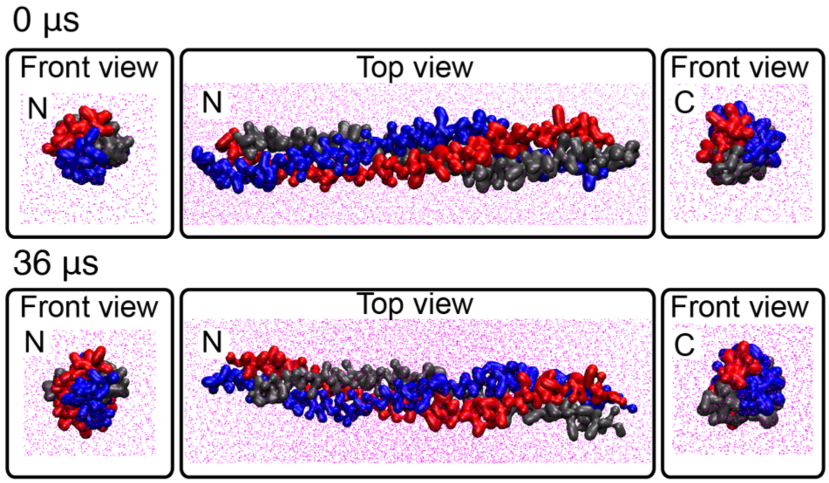
0 µs
Front view
N
Top view
N
Front view
C
36 µs
Front view
N
Top view
N
Front view
C

E

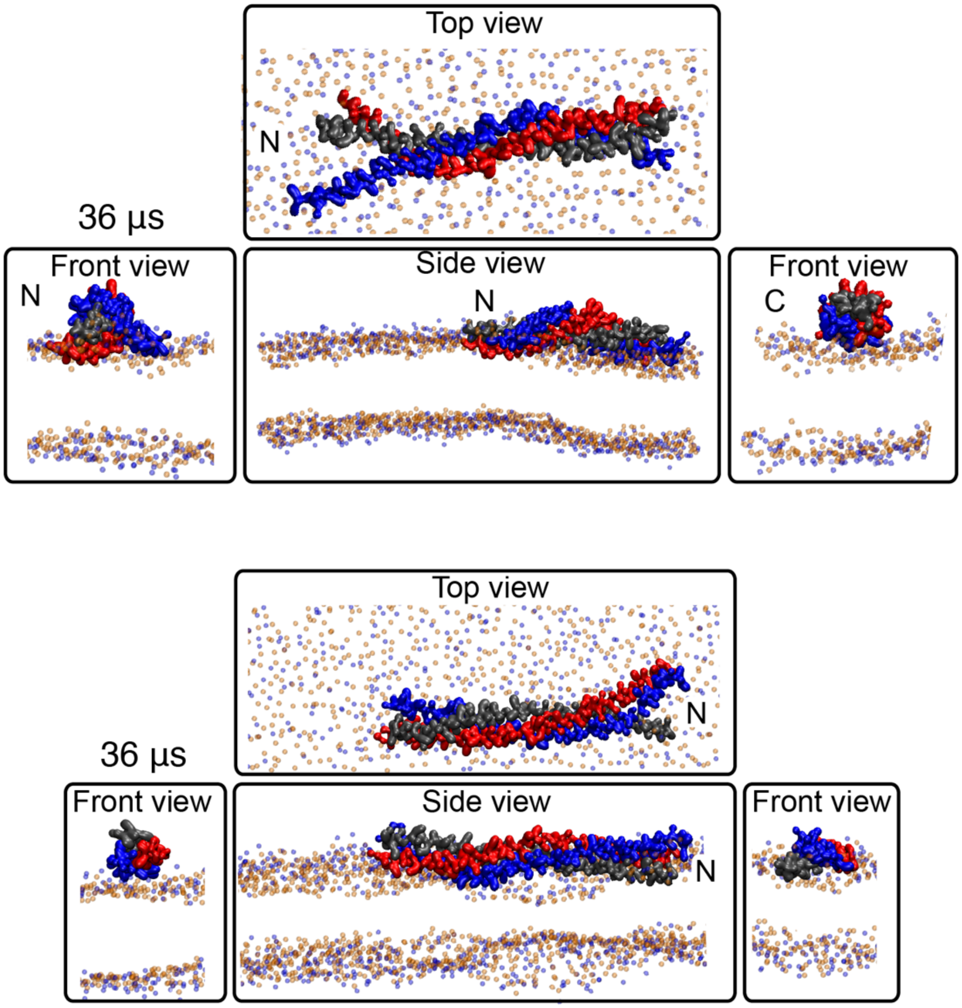
Top view
N
36 µs
Front view
N
Side view
N
Front view
C
Top view
N
36 µs
Front view
Side view
N
Front view

Fig. S7. Molecular dynamics simulations of the binding and orientation of the HR1 domain at the membrane interphase. (A) Coarse-grained molecular dynamics (CG-MD) simulations of HR1 domain binding to membranes consisting of DOPC (silver) and DOPE (green). Helices A, B and C of the HR1 crystal structure are color-coded as blue, red and gray, respectively. Lipid head group beads are shown in blue and orange. **(B)** Snapshots of bottom-up view from membrane toward protein-membrane interphase of trimeric HR1 domain in six CG-MD simulations. Membranes consisted of DOPC, DOPE and PI(4,5)P_2. Helices A, B and C of the HR1 crystal structure are color-coded as blue, red and gray, respectively. **(C)** Illustration of the calculation procedure of membrane thickness. The membrane lipid headgroup beads are represented in blue and brown. The black beads represent the average position of lipid headgroups in outer and inner leaflet for which the thickness is calculated. The green beads represent the lipids surrounding black beads. The arrow on the right panel indicates the local normal for which inner leaflet distance is estimated. **(D)** Snapshots from 0 μs and 36 μs (upper and lower panel, respectively) from different viewing angle from the 36 μs-long simulations of protein trimer in water only. (E) The snapshots of the protein-membrane interaction from different viewing angles shown at 36 μs for two different 36 μs-long simulations of protein trimer interaction in the presence of membrane (upper and lower panel respectively).

Supplementary references

1. V. L. Kuzmin, V. P. Romanov, A. V. Mikhailov, Light-Reflection on the Boundary of Liquid-Systems and Surface-Layer Structure. *Opt Spektrosk+* **73**, 3-47 (1992).
2. C. Schwieger, B. Chen, C. Tschierske, J. Kressler, A. Blume, Organization of T-Shaped Facial Amphiphiles at the Air/Water Interface Studied by Infrared Reflection Absorption Spectroscopy. *J. Phys. Chem. B* **116**, 12245-12256 (2012).
3. J. E. Bertie, M. K. Ahmed, H. H. Eysel, Infrared intensities of liquids. 5. Optical and dielectric constants, integrated intensities, and dipole moment derivatives of water and water-d2 at 22.degree.C. *J. Phys. Chem.* **93**, 2210-2218 (1989).
4. A. Barth, C. Zscherp, What vibrations tell about proteins. *Q. Rev. Biophys.* **35**, 369-430 (2002).
5. T. Buffeteau *et al.*, Anisotropic Optical Constants of α-Helix and β-Sheet Secondary Structures in the Infrared. *J. Phys. Chem. B* **104**, 4537-4544 (2000).
6. M. Rabe, C. Schwieger, H. R. Zope, F. Versluis, A. Kros, Membrane Interactions of Fusogenic Coiled-Coil Peptides: Implications for Lipopeptide Mediated Vesicle Fusion. *Langmuir* **30**, 7724-7735 (2014).

7. S. J. Marrink, H. J. Risselada, S. Yefimov, D. P. Tieleman, A. H. de Vries, The MARTINI Force Field: Coarse Grained Model for Biomolecular Simulations. *J. Phys. Chem. B* **111**, 7812-7824 (2007).
8. S. J. Marrink, A. H. de Vries, A. E. Mark, Coarse Grained Model for Semiquantitative Lipid Simulations. *J. Phys. Chem. B* **108**, 750-760 (2004).
9. O. Kovtun *et al.*, Structural insights into the organization of the cavin membrane coat complex. *Dev Cell* **31**, 405-419 (2014).
10. E. L. Wu *et al.*, CHARMM-GUI Membrane Builder toward realistic biological membrane simulations. *J. Comput. Chem.* **35**, 1997-2004 (2014).
11. H. Bhatia, H. I. Ingólfsson, T. S. Carpenter, F. C. Lightstone, P.-T. Bremer, MemSurfer: A Tool for Robust Computation and Characterization of Curved Membranes. *J. Chem. Theory Comput.* **15**, 6411-6421 (2019).
12. Y. Zhou *et al.*, Caveolin-1 and cavin1 act synergistically to generate a unique lipid environment in caveolae. *J. Cell Biol.* **220**, e202005138 (2021).
13. D. H. de Jong *et al.*, Improved Parameters for the Martini Coarse-Grained Protein Force Field. *J Chem Theory Comput* **9**, 687-697 (2013).
14. W. Humphrey, A. Dalke, K. Schulten, VMD: visual molecular dynamics. *J Mol Graph* **14**, 33-38, 27-38 (1996).
15. S. Buchoux, FATSLiM: a fast and robust software to analyze MD simulations of membranes. *Bioinformatics* **33**, 133-134 (2017).
16. J. Huang, A. D. MacKerell Jr, CHARMM36 all-atom additive protein force field: Validation based on comparison to NMR data. *J. Comput. Chem.* **34**, 2135-2145 (2013).
